# High isolation circularly polarized in-band full-duplex anisotropic dielectric resonator antenna

**DOI:** 10.1038/s41598-023-31159-w

**Published:** 2023-04-12

**Authors:** Mohammad Abedian, Mohsen Khalily, Fan Wang, Pei Xiao, Rahim Tafazolli, Ahmed A. Kishk

**Affiliations:** 1 Institute for Communication Systems (ICS), Home of 5GIC and 6GIC, University of Surrey, Guildford, GU2 7XH UK; 2 Wireless Technology Lab, Huawei Technologies Co., Ltd., No.2222, Xin Jinqiao Road, Shanghai, China; 3 Department of Electrical and Computer Engineering, Concordia University, Montreal, QC H3G 2W1 Canada

**Keywords:** Engineering, Electrical and electronic engineering

## Abstract

A novel high-isolation, monostatic, circularly polarized (CP) simultaneous transmit and receive (STAR) anisotropic dielectric resonator antenna (DRA) is presented. The Proposed antenna is composed of two identical but orthogonally positioned annular sectoral anisotropic dielectric resonators. Each circularly polarized (CP) resonator consists of alternating stacked dielectric layers of relative permittivities of 2 and 15 and is excited by a coaxial probe from the two opposite ends to have left and right-hand CP. Proper element spacing and a square absorber are placed between the resonators to maximize Tx/Rx isolation. Such a structure provides an in-band full-duplex (IBFD) CP-DRA system. Measurement results exhibit high Tx/Rx isolation better than 50 dB over the desired operating bandwidth (5.87–5.97 GHz) with a peak gain of 5.49 and 5.08 dBic for Ports 1 and 2, respectively.

## Introduction

In-band full-duplex (IBFD) devices simultaneously transmit and receive signals at the same frequency^1^. The transmit and receive chains must be isolated significantly to make full-duplex communications possible. According to a link budget calculation, the transmit and receive chains will need to be isolated by at least 110 dB, depending on the application. In other words, multilayer separation and cancellation, self-interference cancellation (SIC), approaches such as antenna isolation, active/passive analog^2^, cancellation circuits, and digital cancellation algorithms^3^ are needed to minimize coupling in a full-duplex system (including antenna, analog, and digital layers). Therefore, to alleviate the requirements of the subsequent self-interference cancellation step, it is critical to take advantage of antennas with high isolation between transmit and receive ports. Since improving SIC in analog and digital domains is challenging, achieving high isolation in the antenna layer reduces the burden placed on other layers and helps mitigate the overall complexity of a STAR transceiver^4^. For example, achieving more than 50 dB isolation in the antenna layer can effectively reduce the burden of analog and digital layers by obtaining a 30 dB isolation for each to overcome the isolation problem in the FD system.

Several techniques have been introduced to enhance the isolation in the full-duplex antenna, such as antenna elements spatial separation^5^, near-field cancellation^6,7^, characteristic modes^8,9^, and using circulators^10,11^. In addition, based on orthogonal-polarization approaches, different polarizations for Rx and Tx antennas are utilized to improve the inter-port isolation^6,12–18^. Full-duplex CP antennas with identical radiation characteristics have recently received significant attention^6,14–18^. Although these antennas show good performance, they have a complex structure and low radiation efficiency^14–16,18^. Also, inter-port isolation is unstable^6^, and large distance between elements^17^.

In the past few decades, CP antennas have been extensively employed in many practical applications like satellite communication to enhance the system performance by offering orientation angle flexibility and mitigating the multipath propagation and fading effects between receiving and transmitting antennas^19^. Here, we propose a highly isolated circularly polarized IBFD dielectric resonator antenna (DRA) for C-band applications with high radiation efficiency for satellite communications. DRA is employed in this structure due to its extraordinary features, such as geometrical flexibility, no surface wave, and high radiation efficiency^20–22^. Furthermore, the 3-D nature of DRAs provides a higher degree of freedom to use height and shape as design parameters over printed antennas^22,23^. The proposed new high-isolation CP IBDF antenna with left-hand CP (LHCP) and right-hand CP (RHCP) for Rx and Tx comprises two orthogonal CP anisotropic annular sectoral dielectric resonators (AAS-DRs) excited by a coaxial probe attached to a rectangular metal strip. Each resonator is composed of staked annular sectoral layers alternating with relative permittivities of 2 and 15. Such an arrangement provides an anisotropic equivalent dielectric. It is worth mentioning that the proposed antenna design helps us tackle the bottleneck problems associated with the existing full-duplex systems by completely bypassing the analog self-interference circuitry.

**Figure 1:**
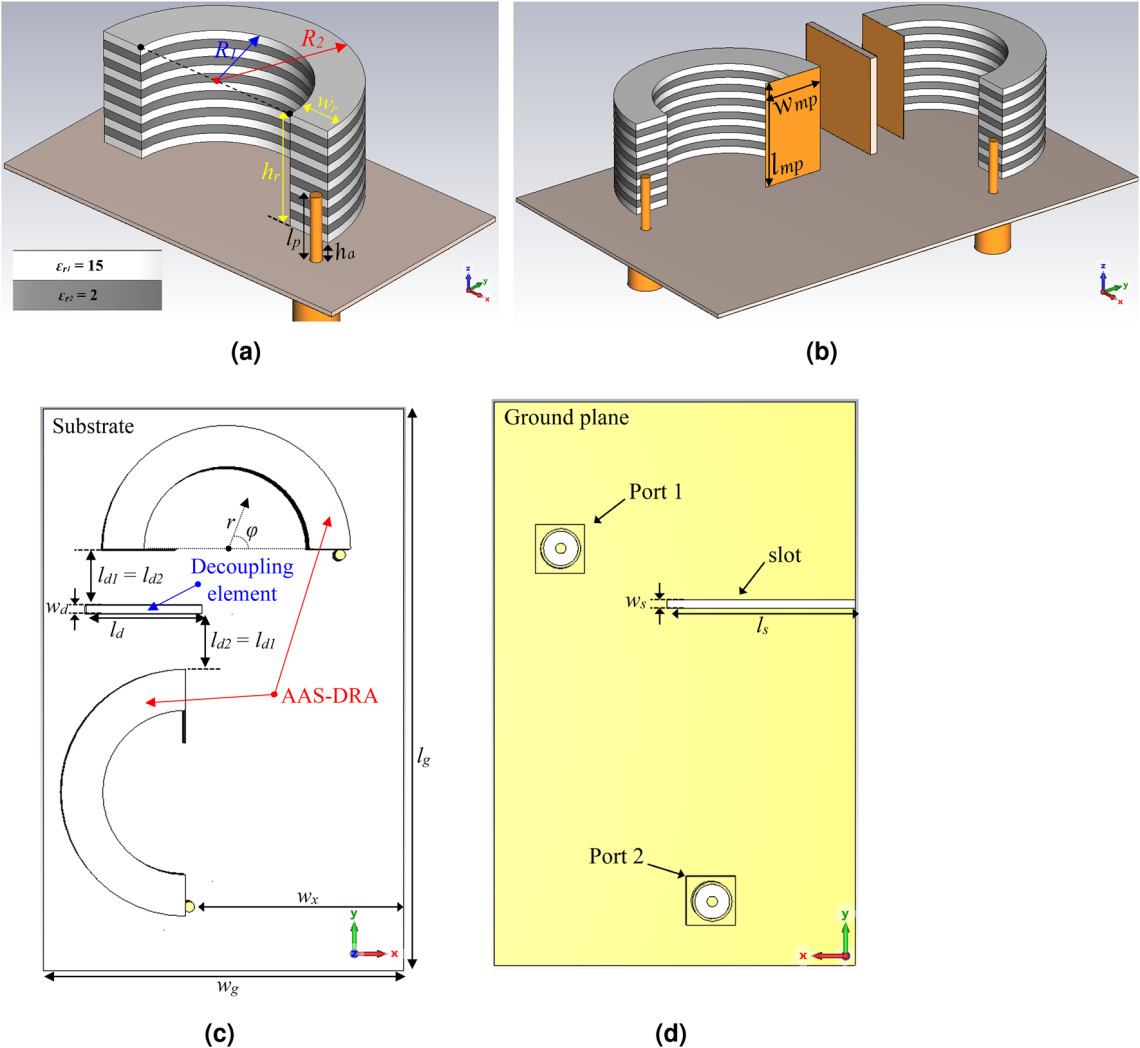
Geometry of the proposed antenna: (**a**) single, (**b**) IBFD (3D view), (**c**) IBFD (top view), and (**d**) IBFD (back view) with $l_g = 69$, $w_g = 44$, $l_{mp} = 15$, $l_p = 7.5$, $l_s = 23$, $l_d = 14$, $l_{d1} = l_{d2} = 6.8$, $w_{mp} = 9$, $w_s = 1$, $w_d = 1$, $w_x = 26.4$, $w_r = 5$, $h_1 = h_2 = 1$, $h_r = 13$, $h_d = 14$, $h_a = 2$, $R_1 = 10$, $R_2 = 15$, $\varepsilon _{r_1} = 15$, $\varepsilon _{r_2} = 2$, $\varepsilon _s = 3.55$. All dimensions in mm.

## Antenna configuration and physical working mechanism

The proposed CP IBFD AAS-DRA shares the same antenna radiation elements and physical aperture. The geometry of the proposed antenna system is illustrated in Fig. 1, including two annular sectors DRs orthogonally oriented and excited from two opposite ends to generate right-hand circular polarization (RHCP) and left-hand circular polarization (LHCP). Each resonator comprises 13 annular sector layers of staked alternating dielectric of relative permittivity 2 (6 layers) and 15 (7 layers) to create a CP-DRA with enhanced AR bandwidth. The thickness of each layer is $$h_1 = h_2 = 1 $$ mm with a relative loss tangent $$tan\, \delta $$ = 1.54 $$\times $$ $$10^{-5}$$. A grounded 44 mm $$\times $$ 69 mm Rogers RO3003 substrate with a relative permittivity of $$\varepsilon _s$$ = 3.55 and a thickness of *s* = 0.508 mm is used to support the resonators. Two coaxial probes are separately connected to each resonator through a vertical metal strip. There is an air gap beneath each DR with a thickness of 2 mm to improve the impedance bandwidth. A rectangular metal plate is vertically attached to the annular sector side with no coaxial probe as a short-circuited end to enhance the CP bandwidth further. A dielectric substrate with relative permittivity of 10 and thickness of 1 mm with conductor cladding on both sides is placed vertically between the short circuit ends of the resonators. CST Microwave Studio 2021 is employed to analyze and optimize the proposed CP antenna.

**Figure 2:**
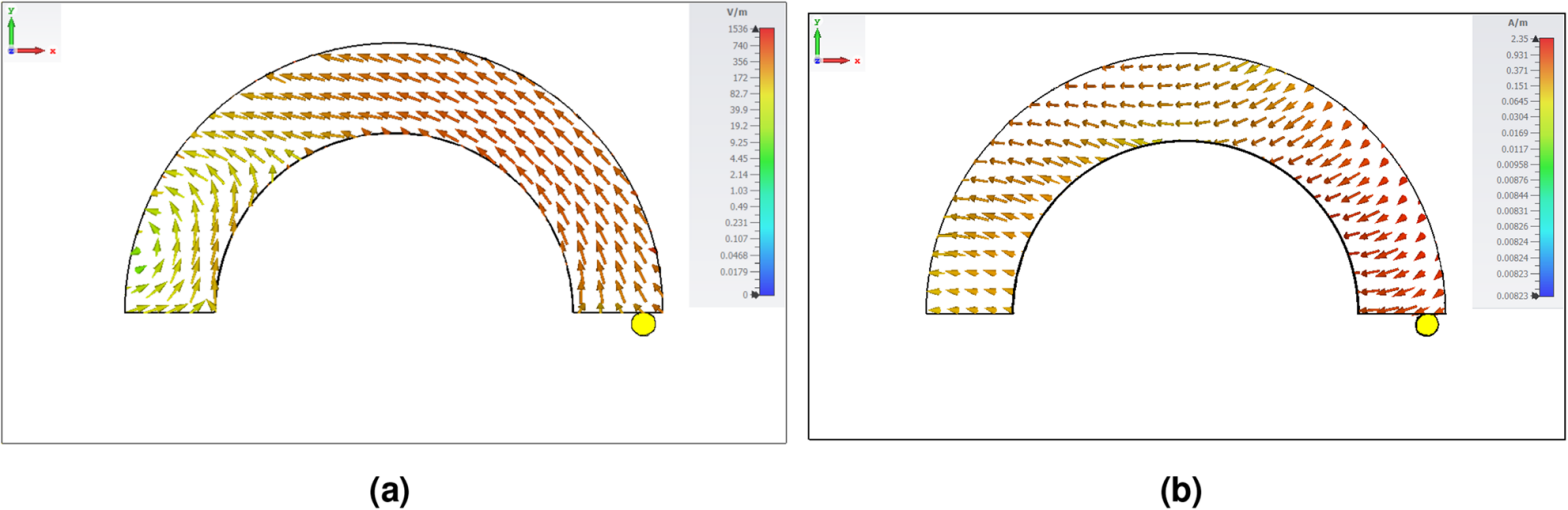
Modes of the AAS- DRA. (**a**) E-field of $TE_{111}^z$ mode and (**c**) H-field of $TM_{111}^z$ mode.

**Figure 3:**
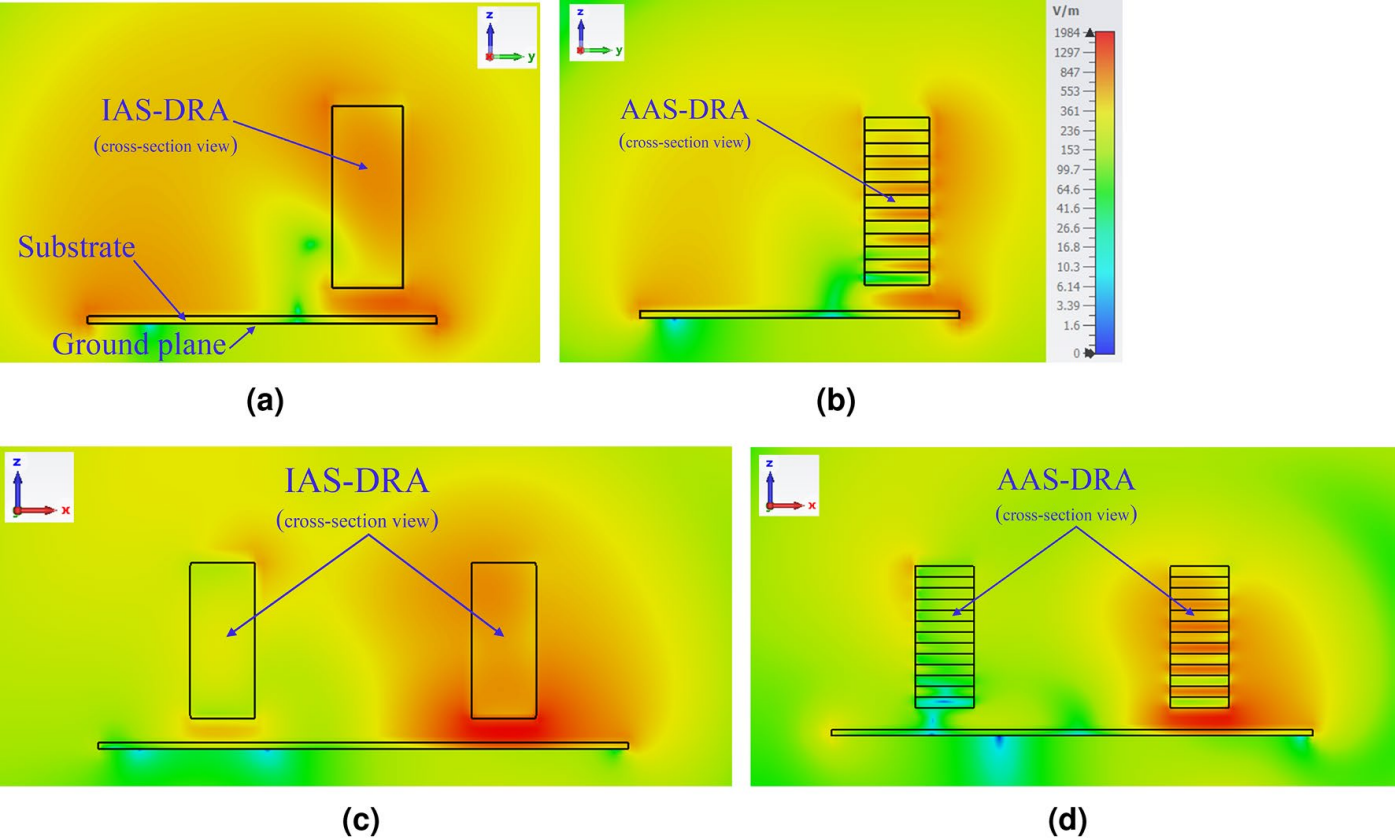
Simulated electric field distribution of annular sector DR. (**a**) IAS (*x*-direction), (**b**) AAS (*x*-direction), (**c**) IAS (*y*-direction), and (**d**) AAS (*y*-direction) at 6.3 GHz.

### Single CP AAS-DRA design analysis

As shown in Fig. 1a, a circularly polarized AAS-DRA is designed based on an isotropic annular sector DRA (IAS-DRA) formed by equivalently removing a sector of an annular cylinder dielectric material. The LHCP and RHCP are realized by placing the coaxial probe feed at the right or left sides of the center, along the radial direction. In other words, considering the terms sin($\phi$) and cos($\phi$) in the electric field expression, RHCP or LHCP fields are excited for broadside radiation when the coaxial probe is placed on the right or left sides of the center, respectively. The semi-cavity model^24^ analyzes the fields inside the annular sector DRA to predict the resonant frequency. The ground plane is defined as an infinite perfect electrical conductor (PEC) plane so that the image theory is utilized. Thus, the stacked DR of double the height is placed inside the waveguide of perfect magnetic conducting (PMC) walls of the annular sector radii $R_1$ and $R_2$, and the end side at $\phi = 180^\circ$ is considered perfect electric conductor (PEC). The top and the bottom of the double-height DR are dielectric. The rest of the waveguide is filled with air. Therefore, standing waves are considered inside the DR, and evanescent waves outside the DR. This is referred to as the dielectric waveguide model, which supports Hybrid modes^22^. However, for simplicity, the top is considered PMC. Thus, the structure becomes a cavity supporting $TE^z$ and $TM^z$ modes, as shown in Fig. 2. Based on these approximate boundary conditions, the first resonant modes are $TE_{110}^z$ and $TM_{111}^z$. Going back to the original height of the DR, the bottom is considered a PEC. Then, the $TE_{110}^z$ is suppressed, and the $TE_{111}^z$ is supported.

An anisotropic annular sector DRA is realized by periodically stacking seven layers of dielectric 1 ($$\varepsilon _{r1}$$ = 15, $$d_1 = 1 $$ mm) and six layers of dielectric 2 ($$\varepsilon _{r2}$$ = 2, $$d_2 = 1$$ mm), where each sheet’s thickness is small enough (less than one-tenth of the wavelength). The overall stacked structure behaves like a homogeneous anisotropic medium, where the equivalent homogenized relative permittivity tensor parameters ($$\varepsilon _x = \varepsilon _y > \varepsilon _z$$) are given by^25^:

1 $$\begin{aligned} {\bar{\bar{\varepsilon }}_{eq}} = \begin{bmatrix} 8.5 &{} 0 &{} 0\\ 0 &{} 8.5 &{} 0\\ 0 &{} 0 &{} 3.53 \end{bmatrix} \end{aligned}$$

Figure 3 illustrates the electric field distribution of the corresponding $TE_{111}^z$ mode for the AAS-DRA and the IAS-DRA at 6.3 GHz. It can be observed from Fig. 3a that compared to the top wall of the AAS-DRA, the electric field is significantly distributed over the side wall. However, as for the IAS-DRA, the electric field magnitudes are almost the same over the side walls and top wall of the IAS-DRA, as shown in Fig. 3b. In other words, by utilizing the anisotropic medium, where the $\varepsilon _x = \varepsilon _y-to-\varepsilon _z$ ratio is higher than one, the electric field magnitude in the z-direction is enhanced, increasing the antenna’s directivity.

**Figure 4:**
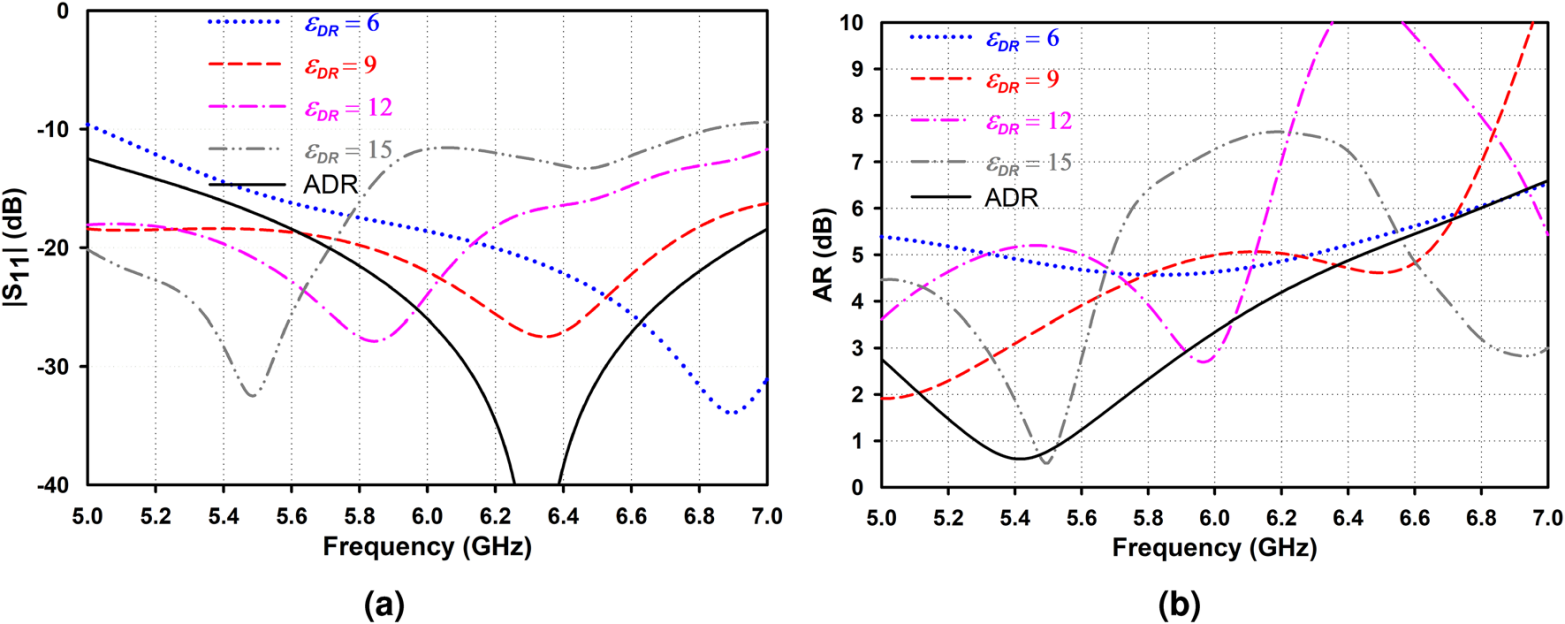
Simulated (**a**) $|S_{11}|$ and (**b**) AR of the IAS- and AAS-DRAs.

**Figure 5:**
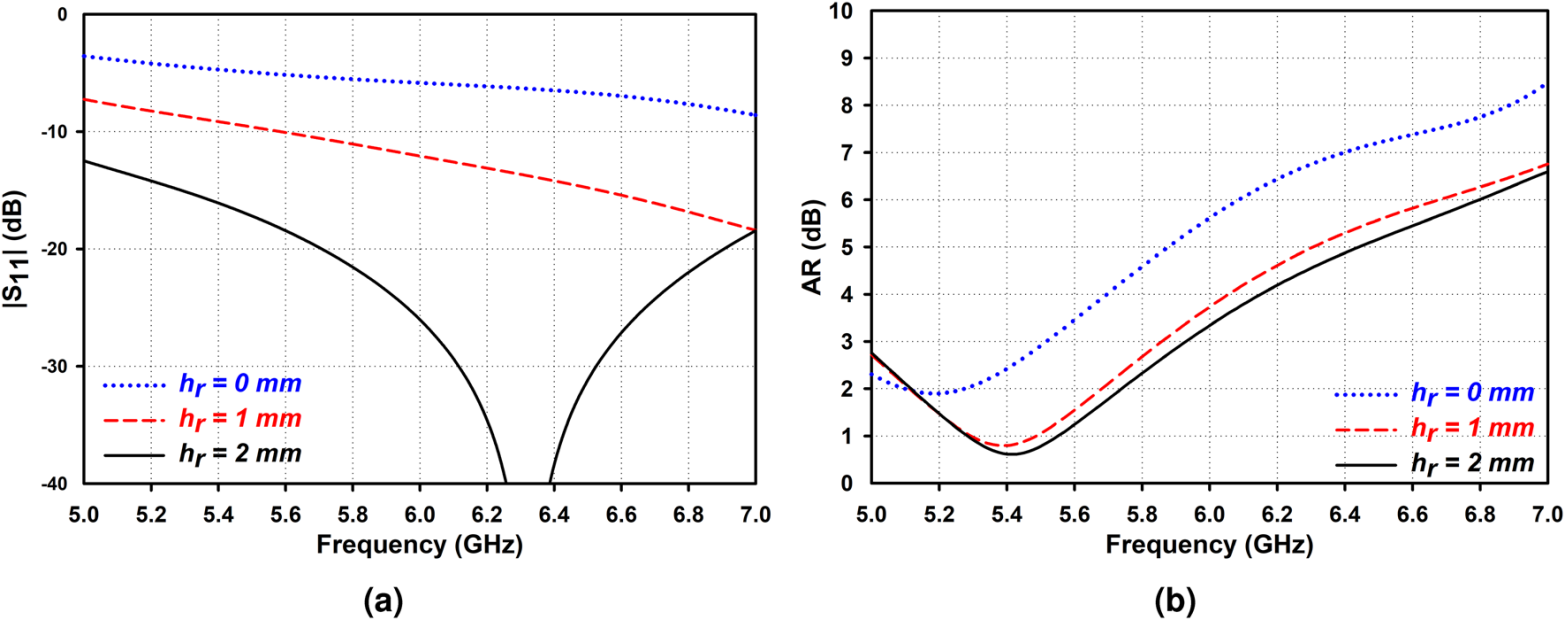
Simulated (**a**) $|S_{11}|$ and (**b**) AR of the AAS-DRA with different values of air gaps.

To achieve CP performance, a coaxial probe is attached to the right sector face of AAS-DRA with a distance from the outer edge of about 1 mm, demonstrating RHCP characteristics. Considering the proposed single CP antenna design, isotropic annular sector DRA with four dielectric constants of $\varepsilon _{r}$ = 6, 9, 12, and 15 are compared with the proposed antenna in the $|S_{11}|$ and AR, as shown in Fig. 4. It can be seen from the figure that the proposed antenna offers a wider 3-dB AR bandwidth with overlapping impedance bandwidth due to producing degenerate resonant modes, which increases the cross-polarization ratio that is desirable for circular polarization designs^25^. In addition, an air gap, $h_a = 2$ mm, is added beneath the DRs to enhance the impedance bandwidth. As shown in Fig. 5, increasing the air gap to $h_a = 2$ mm, the impedance bandwidth significantly improves due to lower effective permittivity.

**Figure 6:**
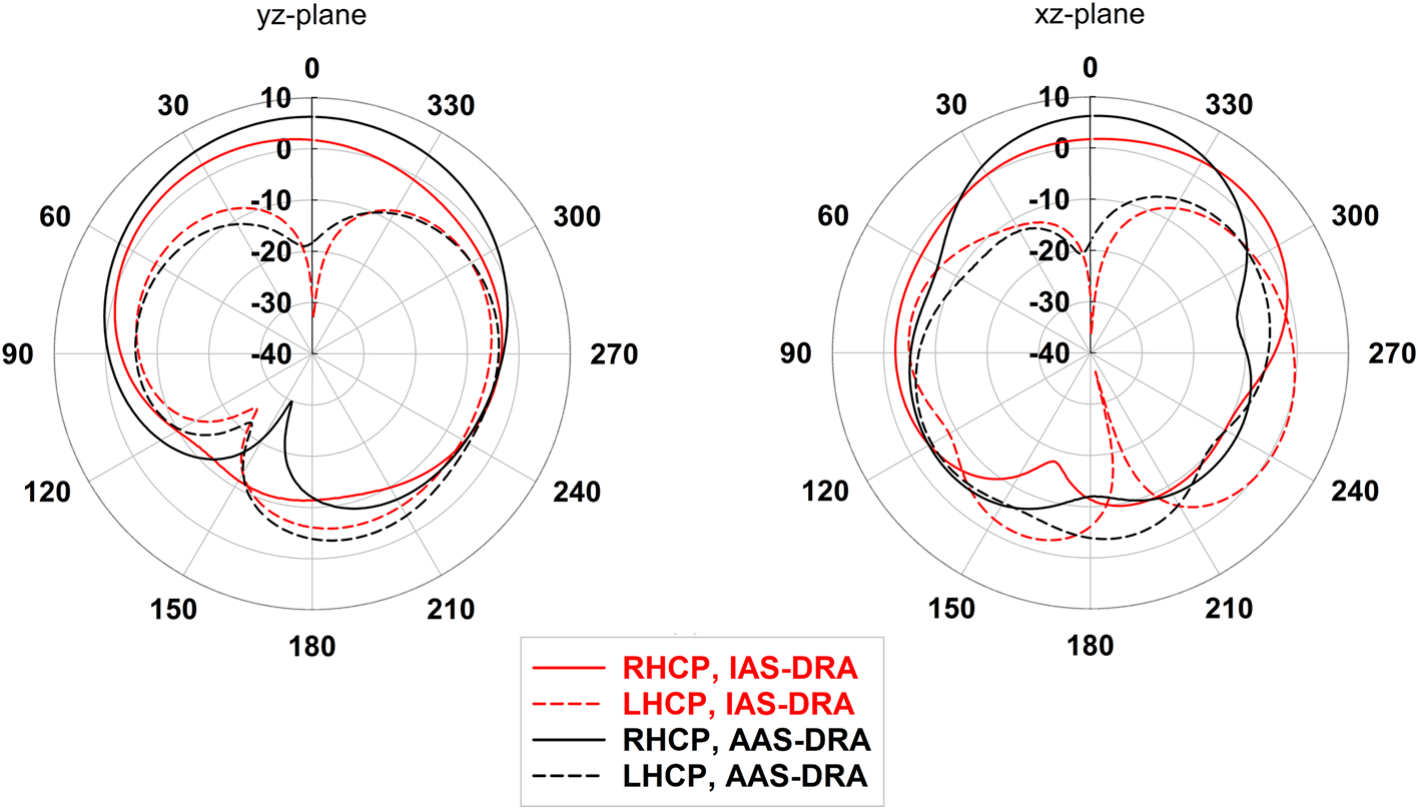
Simulated radiation patterns of the IAS-DRA and the AAS-DRA in the *xz*-plane (left) and *yz*-plane (right) at 5.5 GHz.

Figure 6 illustrates simulated H- and E-plane radiation patterns of the IAS-DRA ($\varepsilon _{r} = 15$) and AAS-DRA at 5.5 GHz, where the AR is minimum. The total radiation pattern is achieved by considering the sum of the radiation patterns on the side, top, and bottom walls. Consequently, using the anisotropic medium improves the intensity of the side wall radiation compared to the top and bottom walls leading to an enhancement of the total directivity in the boresight direction.

### IBFD CP AAS-DRA design analysis

**Figure 7:**
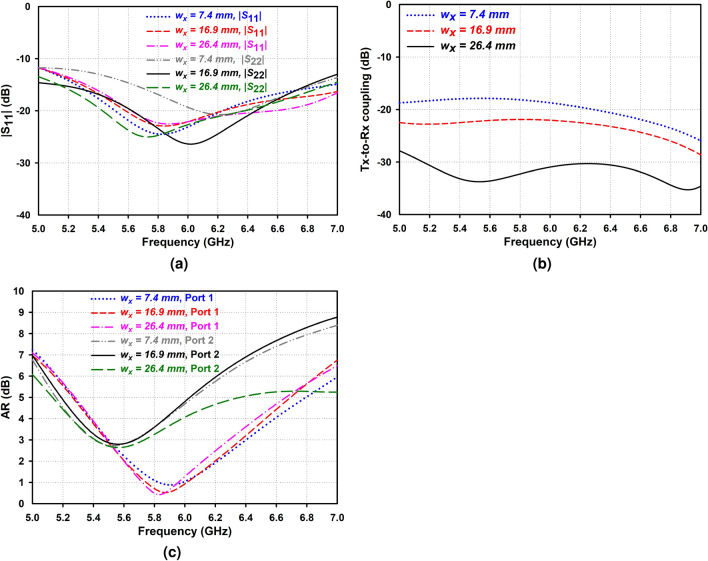
Effect of changing $w_x$ on (**a**) $|S_{11}|$, (**b**) isolation, and (**c**) AR.

As shown in Fig. 1b, two identical CP AAS-DRA are orthogonally excited by coaxial probes, but the opposite sector faces to realize the RHCP and LHCP performance. The distance between Rx and Tx (center-to-center) is 0.63 $\lambda$, and the system input (Port 1) and output (Port 2) ports of the proposed antenna can simultaneously operate. Figure 7 illustrates the antenna’s simulated $|S_{11}|$, isolation, and AR when the second annular sector AAS-DRA moves along the x-direction with a key parameter $w_x$. This figure shows that the antenna isolation is sufficiently improved by choosing a proper value of $w_x = 26.4$ mm, but changing $w_x$ has a minor effect on the $|S_{11}|$ and AR. It is found that a 3-dB axial ratio related to the second port, from 5.41 to 5.74 GHz, is obtained, which could be enhanced further. It is noted that various arrangements of the two orthogonally polarized annular sector DRs can achieve more compactness. However, these arrangements will not achieve the desired high isolation.

**Figure 8:**
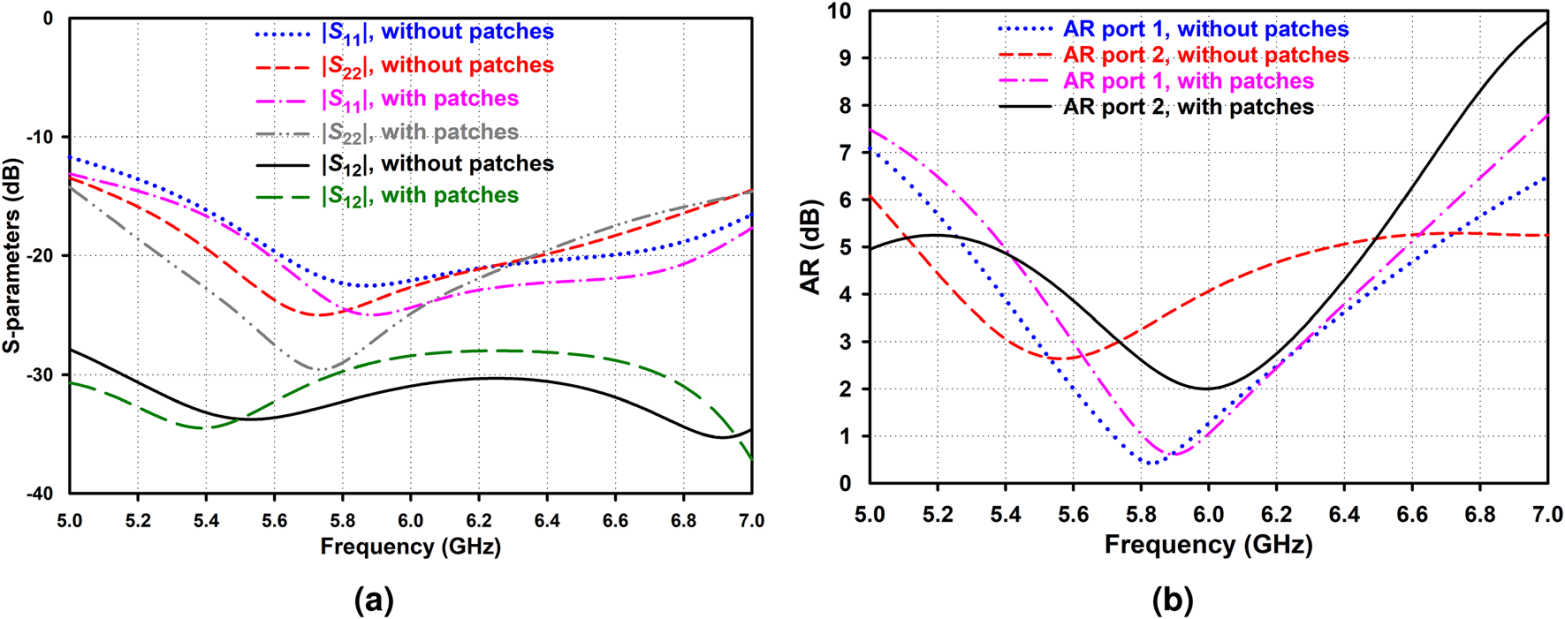
Simulated (**a**) S-parameters and (**b**) AR of the proposed antenna with and without the metal patches.

In order to enhance the CP bandwidth, two rectangular short-circuited plates of equal dimensions are attached to the left and right sector faces of each AAS-DRA, respectively (see Fig. 1b). Figure 8 depicts the S-parameter and AR with and without patches. It can be seen from Fig. 8b that by adding the metal patches, the 3-dB AR bandwidth related to the second port improves from 330 to 520 MHz.

**Figure 9:**
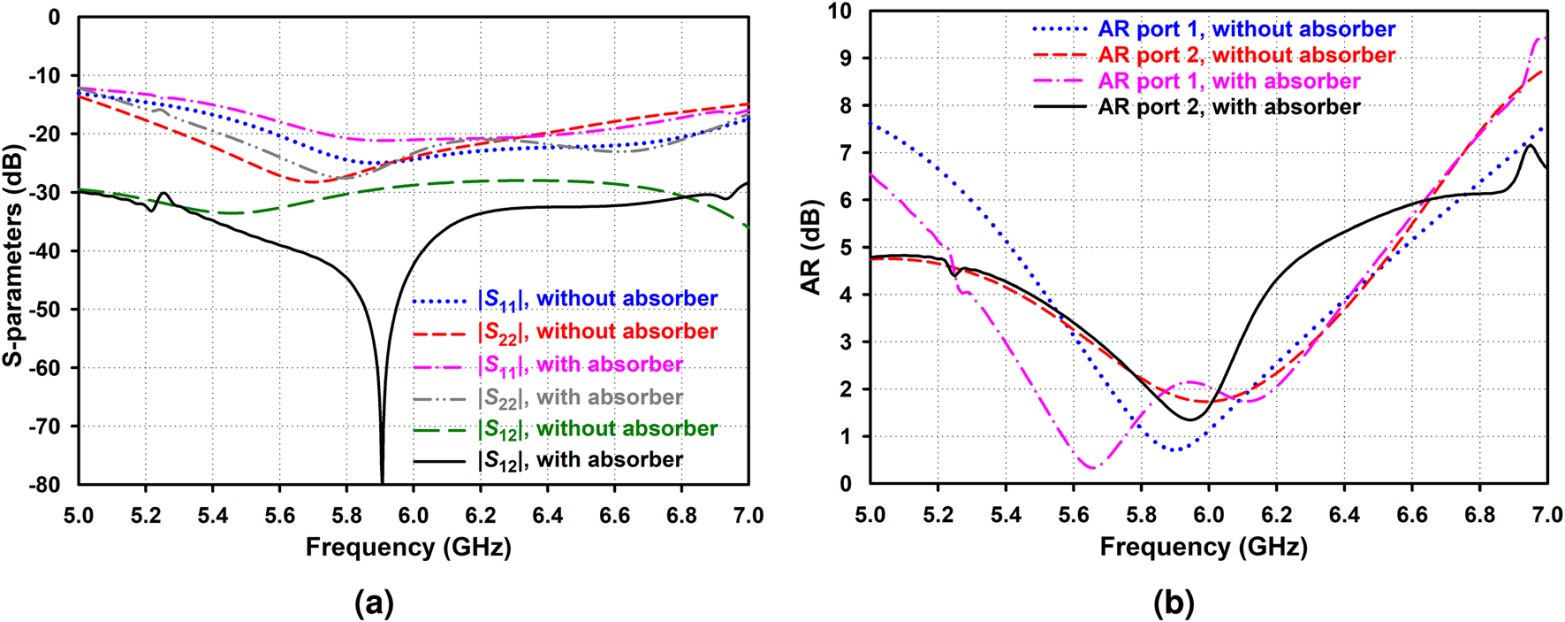
Simulated (**a**) S-parameters and (**b**) AR of the proposed antenna with and without the absorber.

Finally, since the main aim of this work is to propose a new circularly polarized IBFD antenna with high isolation of over 50 dB, a decoupling structure is employed to increase the antenna isolation. First, a slot is etched in the ground plane (see Fig. 1d) to reduce the ground plane effect. Then, as illustrated in Fig. 9, by adding a dielectric slab with dielectric constant 10, covered by two identical metal patches from the two sides, in the middle of two AAS-DRAs, at the top of the slot, on the substrate, the mutual coupling between Rx and Tx is significantly reduced. This phenomenon arises due to reducing the scattering waves between two resonators, especially around the absorber, utilizing high impedance characteristics at the edges of the absorber, leading to substantially enhanced isolation between the Tx and Rx ports with an isolation bandwidth ranging from 5.85 to 5.95 GHz over 50 dB.

## Experimental results

**Figure 10:**
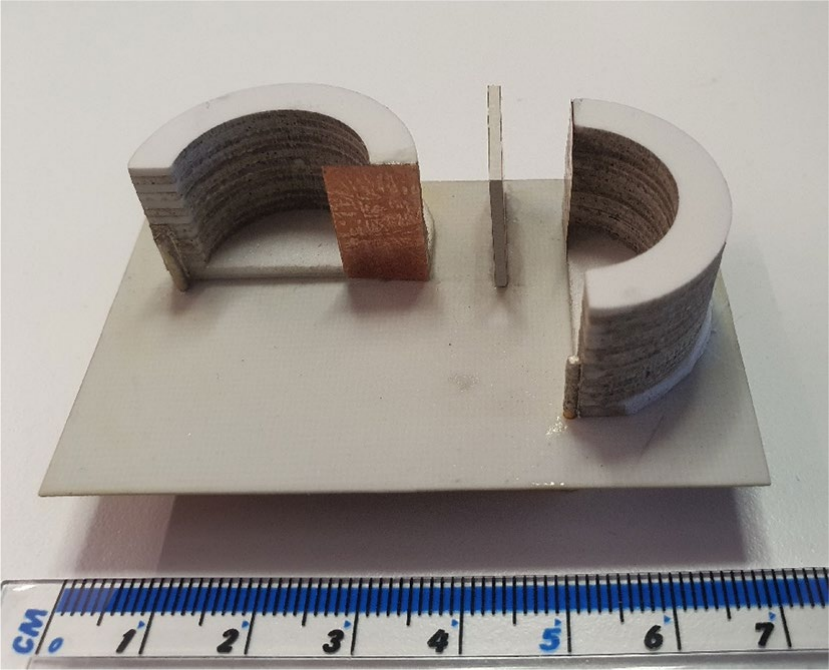
Implemented prototype of the proposed antenna.

As shown in Fig. 10, a prototype of the proposed antenna is fabricated and measured, verifying the performance of the proposed antenna. A ROHACELL® HF Foams ($\varepsilon _{ro}$ = 1.04) is glued beneath each lower DR using RTV silicone adhesive ($\varepsilon _g \approx 3$) as an air gap.

### S-parameter measurement

**Figure 11:**
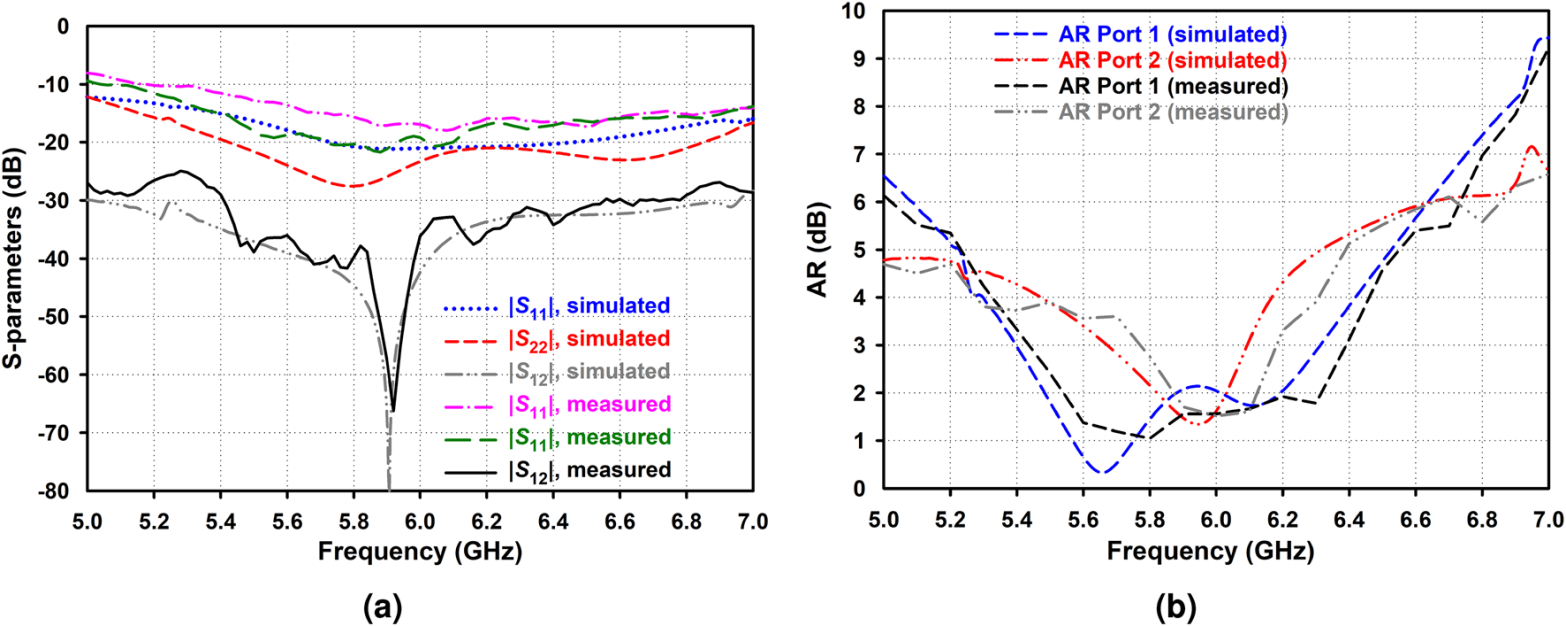
Simulated and measured (**a**) S-parameters and (**b**) ARs of the proposed antenna.

The simulated and measured S-parameters and ARs of the proposed antenna are illustrated in Fig. 11, indicating a reasonable agreement between the simulated and measured results except for a trivial discrepancy caused by the fabrication errors and manually assembling imperfections. The proposed CP IBFD AAS-DRA provides a perfectly overlapping impedance bandwidth for ports 1 and 2. As shown in Fig. 11a, the proposed antenna offers isolation higher than 50 dB in desired bands, ranging from 5.85 to 5.95 GHz and 5.87 to 5.97 GHz for simulation and measurement, with the maximum isolations up to 80.94 dB at 5.88 GHz and 66.25 dB at 5.92 GHz, respectively. As illustrated in Fig. 11b, the proposed antenna provides a measured AR bandwidth from 5.44 to 6.32 GHz and 5.77 to 6.18 GHz for Ports 1 and 2, respectively.

### Far-field measurement

**Figure 12:**
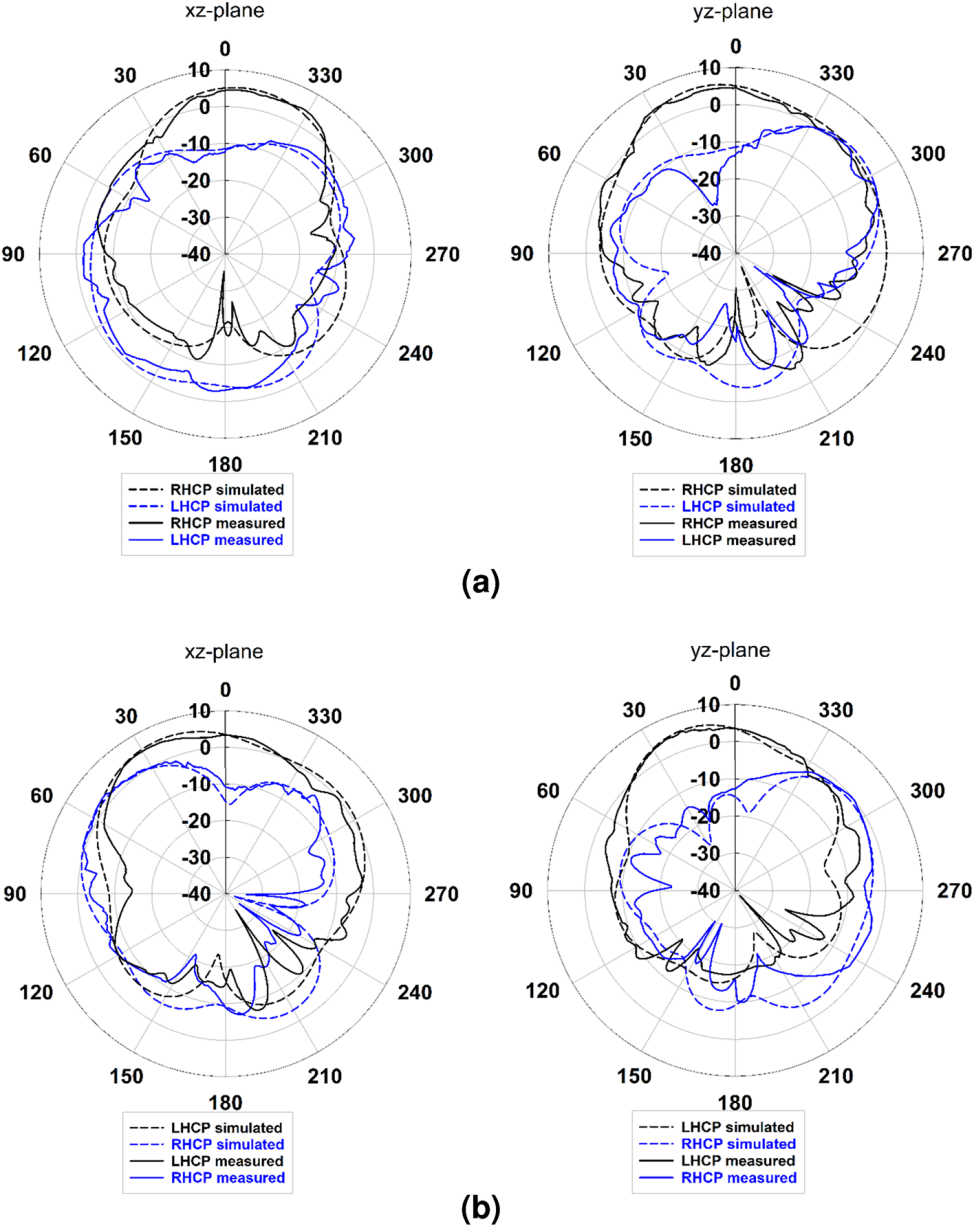
Simulated and measured RHCP and LHCP radiation patterns of the proposed antenna in *xz*-plane (left) and *yz*-plane (right) at 5.9 GHz; (**a**) Port 1 and (**b**) Port 2.

Figure 12 depicts the simulated and measured LHCP and RHCP radiation patterns of the proposed antenna in the *xz*-plane ($\phi = 0^\circ$) and *yz*-plane ($\phi = 90^\circ$) at 5.9 GHz. It can be seen that the simulated and measured patterns are in good agreement. The difference between LHCP and RHCP radiation levels is more than 18 dB, confirming the radiation’s purity. It is worth mentioning that the proposed antenna provides a total simulated efficiency higher than 98% in the desired frequency bands with a maximum simulated realized gain of about 5.93 and 5.56 dBic for Port 1 and Port 2, and measured 5.49 and 5.08 dBic, respectively.

### Comparison with the literature

The main contribution of the proposed work is designing a novel structure to provide a circularly polarized performance for RHCP or LHCP based on the exciting end with high inter-port isolation of two orthogonal CP-DRA within the desired impedance bandwidth, high total efficiency, and almost the same gain levels for both Tx and Rx modes. In order to compare our antenna with other anisotropic antennas published in the literature, it is worth mentioning that in this work, the electric and magnetic fields of the fundamental mode of the AAS-DRA are obtained using a semi-cavity model. This model supports $TE^z$ and $TM^z$ modes to increase the cross-polarization ratio that is necessary for circular polarization design. The proposed antenna is compared with the recent state-of-the-art circularly polarized high isolation antennas in Table 1. The proposed antenna offers high isolation with a total efficiency of about 98% using the monostatic configuration.

**Table 1:** Comparison with CP IBFD antennas in the literature

References	No. of elements	Overlapping BW (MHz)	$|S_{12}|$ (dB)	Tx/Rx peak gain (dBic)	Eff.
^14^	4	100	47	$\ge$ 7	$\ge$ 95
^15^	4	100	41	6.8/7.6	$\ge$ 62
^16^	4 $\times$4	1200	40	$\ge$ 8.7	NA
^6^	4	100	41	7.2/10.5	$\le$ 80
^17^	4	100	70	6.8/6.9	$\le$ 54
^18^	3	75	47	3.4/6.4	$\le$ 51
Present	2	100	50	5.08/5.49	$\ge$ 98

## Conclusion

A circularly polarized in-band full-duplex anisotropic annular sector dielectric resonator antenna has been presented. Two anisotropic annular sector DRs with two different dielectric constants have been employed to generate right-hand circular polarization (RHCP) and left-hand circular polarization (LHCP) to improve the impedance bandwidth with better isolation. In addition, a decoupling structure has been proposed to further isolation enhancement. The measured results have demonstrated high isolation over 50 dB with an overlapping impedance bandwidth with AR bandwidth ranging from 5.87 to 5.97 GHz. Furthermore, the proposed antenna has offered high efficiency and isolation, making it a potential candidate for full-duplex applications.

## Data Availability

The data that support the findings of this study are available from the corresponding author upon reasonable request.
